# Two Years of Revolutionizing Oral Health in Tanzania: Evaluation of a Multicomponent Government-Led Model

**DOI:** 10.1155/ijod/7004986

**Published:** 2025-09-23

**Authors:** Baraka J. Nzobo, Sima Rugarabamu, James Tumaini Kengia, Mavere Tukai, Hamad Nyembea

**Affiliations:** ^1^Oral Health Services, Ministry of Health, Dodoma, Tanzania; ^2^Clinical Research, Training and Consultancy Unit, Muhimbili National Hospital, Dar es Salaam, Tanzania; ^3^President's Office – Regional Administration and Local Government, Dodoma, Tanzania; ^4^Medical Stores Department, Ministry of Health, Dar es Salaam, Tanzania; ^5^Directorate of Curative Services, Ministry of Health, Dodoma, Tanzania

**Keywords:** dental amalgam phase-out, health policy, health system strengthening, oral health reform, primary health care, Tanzania

## Abstract

**Background:** In 2022, the Tanzanian government launched a comprehensive multicomponent reform aimed at revolutionizing the national oral health care system. This initiative is part of a broader commitment to align with global health standards and address the significant oral health challenges faced by the population. Prior to the reforms, Tanzania's oral health infrastructure was marked by outdated practices and high prevalence rates of oral diseases, including dental caries and periodontal disease.

**Methods:** We employed a qualitative case study design, guided by Walt and Gilson's Policy Triangle and Kingdon's Multiple Streams Framework, to analyze the planning, implementation, and early outcomes of the reform. Data were sourced from national surveys, District Health Information System-2 (DHIS2), official policy documents, procurement and training reports, and triangulated across multiple systems. Analysis focused on reform milestones, service utilization trends, and contextual drivers.

**Results:** The reform resulted in significant system-wide changes, including the procurement of 340 modern dental chairs, installation of 306 digital radiography units, and national phase-out of dental amalgam. An Oral Health Scorecard was deployed to monitor restorative treatment trends, contributing to a reduction in the extraction-to-restoration ratio from 2.8:1 in 2022 to 1.3:1 in 2025. The introduction of a pregnancy-focused oral health program and expanded training for dental therapists further enhanced service equity and quality. Reform implementation was enabled by strong political will, professional consolidation, and integration within existing national health strategies.

**Conclusion:** Tanzania's rapid and multicomponent oral health reform represents a pioneering model for low- and middle-income countries. By aligning with the WHO Global Oral Health Action Plan and embedding oral health into primary and maternal healthcare, the country has laid the foundation for sustainable and equitable dental care. Continued investments in digital innovation, public–private partnerships, and health education will be critical for scaling impact.

## 1. Background

Oral health is a vital component of overall well-being, encompassing the health of the mouth, teeth, and orofacial structures, which are essential for fundamental functions such as eating, breathing, and speaking [1]. It also significantly influences psychosocial factors, including self-confidence and the ability to interact socially without pain, discomfort, or embarrassment [1, 2]. In Tanzania, oral diseases share common risk factors with major noncommunicable diseases (NCDs), such as tobacco use, harmful alcohol consumption, and high sugar intake, emphasizing the interconnected nature of oral and general health [3, 4].

Before 2022, Tanzania's oral health infrastructure faced significant challenges [5]. It relied heavily on outdated materials like dental amalgam, valued for its durability but criticized for its mercury content, which posed serious health and environmental risks [5, 6]. This reliance was at odds with international health standards, such as the Minamata Convention on Mercury, which advocates for the phasing down and phasing out of mercury use in healthcare.

The prevalence of oral diseases in Tanzania has been alarmingly high, with 76.5% of adults and 31.1% of children affected by dental caries, while periodontal diseases impact 62.8% of adults and 57.4% of children [7, 8]. These conditions not only cause pain and disability but also lead to significant economic losses due to reduced productivity and the high costs of treatment [8].

In response to these pressing challenges, the Tanzanian government embarked on a comprehensive multicomponent reform of the oral health sector in 2022. This reform included phasing out the use of dental amalgam in favor of safer and more sustainable materials such as glass ionomer cement (GIC), composite resin, and Cention N, aligning with global health standards. Additionally, the government invested in the procurement and installation of advanced dental equipment, including modern dental chairs and digital radiography systems, across healthcare facilities at all levels from national hospitals to primary health care facilities. These initiatives have significantly enhanced the quality of dental care, expanded access to services, and ensured equitable oral health services throughout the country.

This study aims to highlight the progress made in the oral health sector, emphasizing the transformative changes that have improved the quality and accessibility of oral healthcare in Tanzania.

## 2. Methodology

This study employed a qualitative case study design to document and evaluate the multicomponent oral health reforms implemented in Tanzania from 2022 onward. The methodology was guided by a three-step analytical framework adapted from Walt and Gilson's Policy Triangle which considers context, content, actors, and processes and supported by elements of Kingdon's Multiple Streams Framework to interpret policy adoption dynamics.

Our review focused on both implementation and early outcomes of the reform agenda.

The study was structured into three interrelated phases:

Pre-reform contextual assessment: We reviewed the baseline status of Tanzania's oral health system before the 2022 reforms. This involved an in-depth desk review of national oral health policies, the Fifth National Oral Health Survey, routine District Health Information System-2 (DHIS2) data, strategic documents from the Ministry of Health, and reports from Regional and Zonal Dental Officers.

Implementation and progress tracking: Reform components including procurement of equipment, clinical service delivery changes, phase-out of dental amalgam, and scorecard deployment were assessed through triangulation of administrative records, procurement logs, policy briefs, and training program reports. Emphasis was placed on assessing intermediate outputs (e.g., equipment installed and staff trained) and service delivery trends captured in DHIS2 and facility reports.

Contextualization and forward reflection: Data were synthesized thematically and contextualized using Kingdon's framework to explore how the policy window for reform emerged. Reflections were developed to generate practical recommendations for scale-up, sustainability, and regional learning.

### 2.1. Data Sources and Selection Criteria

The data sources for this case study were selected based on their relevance to national oral health programming and policy implementation. Key documents included the Fifth National Oral Health Survey (baseline epidemiology), DHIS2 monthly performance reports (service utilization trend), Regional and Zonal Dental Officer submissions (implementation monitoring), Ministry of Health policy (strategic guidance and mandate), and national procurement data (infrastructure and human resource deployment).

### 2.2. Data Validation and Triangulation

To enhance credibility, we employed methodological triangulation by comparing findings across multiple independent data streams. For example, equipment deployment reported in procurement records was verified against DHIS2 service delivery changes and regional reports. Priority was given to data sources with established quality assurance mechanisms. Deviations were addressed through follow-up interviews with regional officers (informal validation step).

### 2.3. Limitations

The study acknowledges limitations inherent in using administrative data, including potential underreporting, data completeness issues, and regional reporting delays. These were mitigated by using corraborating data from multiple sources, but future prospective evaluations are recommended for a more rigorous impact assessment.

## 3. Results

### 3.1. Procurement of Dental Equipment From 2022 to 2024

The Tanzanian government has made remarkable strides in enhancing oral healthcare infrastructure from 2022 to 2024, significantly impacting the quality and accessibility of dental services nationwide. This ambitious initiative has included the procurement and installation of advanced dental equipment across various healthcare levels, from tertiary hospitals to primary health centers.

A total of 340 state-of-the-art dental chairs have been distributed to healthcare facilities, ensuring improved patient care and comfort. Additionally, 306 digital periapical dental X-ray systems have been installed across the country, enabling precise diagnostics and efficient dental treatments. To cater to complex imaging needs, 18 cutting-edge 3D-cone beam computerized tomography (3D-CBCT) units have been strategically deployed in national, zonal, and regional referral hospitals. Furthermore, 57 digital 2D-orthopantomography units have been set up in district hospitals, significantly expanding access to panoramic dental imaging.

**Figure 1:**
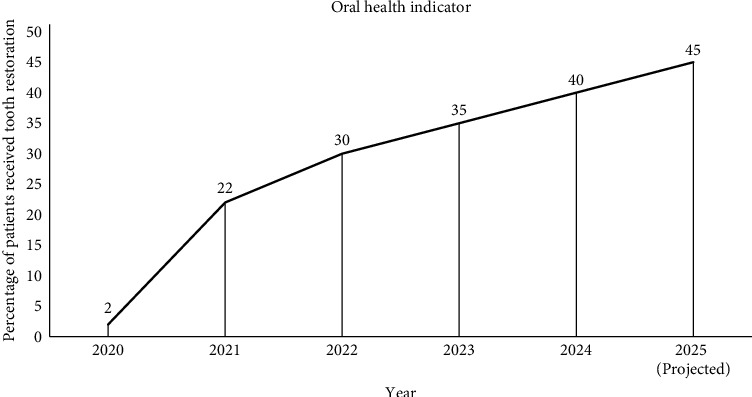
Trends in oral health indicators (2022–2025). The data shows a steady decline in extraction-to-restoration ratios nationally, from 2.8:1 in 2022 to 1.3:1 by mid-2025. The percentage of patients receiving restorative treatments increased from 19% to 47% during the same period.

These advancements are complemented by extensive renovations of public dental clinics to provide a conducive environment for dental practitioners and patients. This comprehensive approach ensures equitable access to high-quality oral healthcare services across all levels of care. Early longitudinal analyses of this initiative indicate a positive impact on oral health outcomes, reflecting the effectiveness of these investments in transforming the dental healthcare landscape in Tanzania (Figure 1).

According to DHIS2, the ratio of tooth extractions to restorative treatments has dropped significantly over the reform period from 2.8 in 2022 to 1.3 by 2025 indicating a shift toward tooth preservation. Similarly, monthly utilization of scaling and root planning services has doubled (12,000–24,800 visits/month) and the national average for digital X-ray-supported diagnoses has increased by 73%.

### 3.2. Phasing Out Dental Amalgam Use in Tanzania

In line with the global commitment to environmentally sustainable health practices, the Ministry of Health has mandated the cessation of dental amalgam use in restorative dentistry across Tanzania. Effective from January 2022, this directive applies to all oral health care providers, encompassing both public and private dental services. The policy extends beyond the initial focus on vulnerable populations, such as pregnant women and children, to include the general population, thereby making Tanzania a leader in adhering to the Minamata Convention of 2013, which aims to phase out dental amalgam use in dental practice [9, 10].

**Figure 2:**
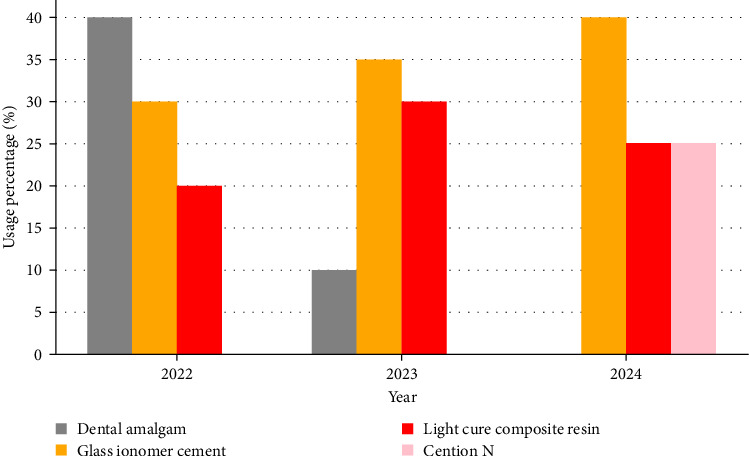
Trends in the use of dental filling materials in Tanzania.

Furthermore, the ministry has prohibited dental practitioners from ordering or purchasing dental amalgam, signaling a definitive move towards phasing it out entirely. In support of this transition, the ministry has conducted extensive capacity-building workshops on alternative dental filling materials. These alternatives, which have been proven to perform comparably to dental amalgam, include GIC, light cure composite resin, and Cention N—the latter being identified as the most effective substitute in Tanzania (Figure 2) [11].

### 3.3. Oral Health Score Card

In Tanzania, oral health services have been systematically integrated into the National Health Management Information System (HMIS) to ensure streamlined data collection and effective monitoring. Public and private dental clinics are mandated by the Ministry of Health to submit routine data from dental treatments on a monthly basis to the DHIS2. This initiative aims to create a robust database for informed decision-making in oral healthcare [12].

To enhance monitoring and evaluation efforts, the ministry has developed an Oral Health Scorecard within the DHIS2 framework. This scorecard tracks key performance indicators, including the proportions of patients receiving tooth restorations (permanent fillings, temporary fillings, and root canal treatments), tooth extractions, scaling procedures, dentures, and cases of infected sockets following extractions. These metrics offer valuable insights into service delivery and patient outcomes, enabling targeted interventions [13].

**Figure 3:**
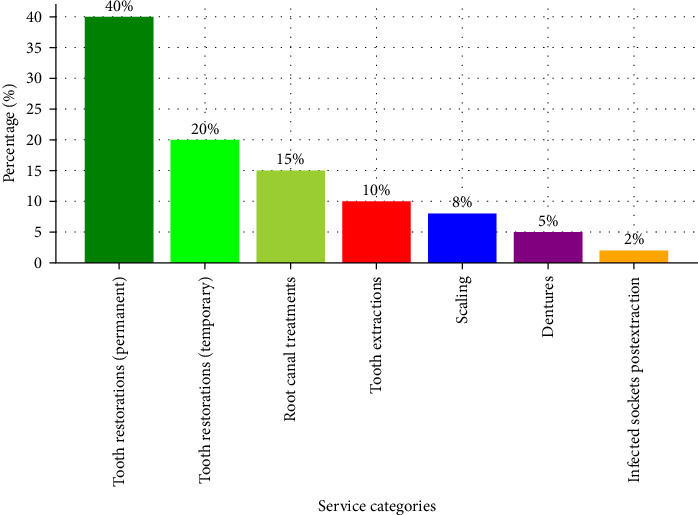
2024 Oral Health Scorecard trends.

The overarching goal of the Oral Health Scorecard is to shift the focus from tooth extractions to restorative treatments, thereby improving the overall dental health of the Tanzanian population. By emphasizing restorations, the ministry aims to preserve natural teeth, reduce the prevalence of dental issues, and promote equitable access to quality oral healthcare services (Figure 3) [7].

### 3.4. Capacity Building to Dental Therapists

In response to the evolving needs of oral health care, the Ministry of Health launched a capacity-building program in 2024 for dental therapists—frontline dental practitioners with diplomas in clinical dentistry [14]. This program, aimed at all dental therapists in primary health care facilities, focuses on the proper use of dental filling materials, operation of digital periapical dental X-rays, execution of single-rooted tooth root canal treatments, utilization of ultrasonic scalers, and enhancement of minor dental surgery skills [15]. This initiative ensures that the majority of Tanzanians receiving care at primary health care facilities experience high-quality dental services.

### 3.5. Oral Health in Pregnancy Program

Understanding the significant connection between oral health and pregnancy outcomes, the Ministry of Health launched the Oral Health in Pregnancy Program in 2022 to address the knowledge gap regarding the importance of dental care during pregnancy. This initiative aims to integrate oral health education and screening into reproductive and child health services across both public and private healthcare sectors.

**Figure 4:**
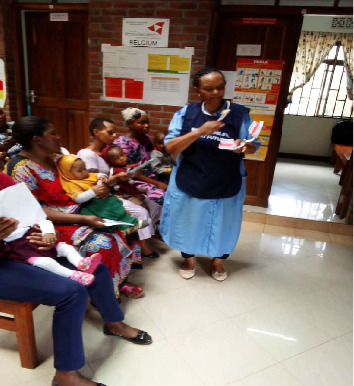
A trained nurse providing oral health education in a reproductive and child health clinic.

As part of the program, nurses and midwives in reproductive and child health clinics are trained to provide oral health education and conduct screenings for pregnant women and children (Figure 4). Individuals identified with dental issues are referred to dental clinics for appropriate treatment. This comprehensive approach ensures early detection and intervention, improving both maternal and child health outcomes.

**Figure 5:**
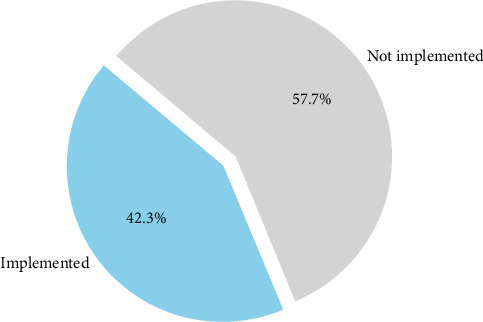
Oral Health in Pregnancy Program: regional implementation.

Currently, the program has been rolled out in 11 of Tanzania's 26 regions (Figure 5), with plans for further expansion contingent on the availability of funding. The initiative highlights the ministry's commitment to improving oral health as an integral part of overall healthcare services for mothers and children.

### 3.6. Enabling Factors for Oral Health Services Improvement

**Figure 6:**
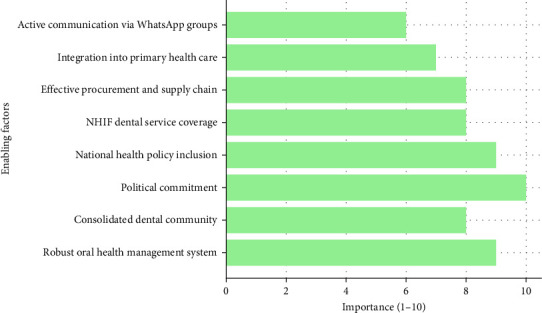
A horizontal bar chart illustrating the importance of various factors contributing to the improvement of oral health services in Tanzania, rated on a scale of 1–10.

The enhancement of oral health services in Tanzania has been underpinned by a constellation of critical factors. Central to these improvements is a robust oral health management system, complemented by the consolidation of the dental community under a singular professional association. This structural evolution has been bolstered by a decisive political commitment from the ruling party, dedicated to elevating the standards of health services across the nation (Figure 6). Further support is seen in the strategic inclusion of oral health within both the National Health Policy and the Tanzania Health Sector Strategic Plan V for the period 2021–2026. The extension of dental service coverage by the National Health Insurance Fund (NHIF) and the effective management of national procurement and supply chains for dental items have significantly enhanced service delivery. Moreover, the integration of oral health services into primary health care facilities, coupled with active communication and collaboration facilitated through dental practitioners' WhatsApp groups, has fostered a more connected and responsive dental health community. These factors collectively contribute to the ongoing improvements and sustainability of oral health services in Tanzania.

### 3.7. Policy Adoption and Implementation Process

The adoption and implementation of Tanzania's oral health reforms can be better understood through the lens of Kingdon's Multiple Streams Framework. This policy model identifies three key streams problems, policies, and politics that must converge to open a window for change.

In Tanzania, the problem stream was defined by alarming oral disease prevalence rates, high reliance on outdated and environmentally hazardous materials such as dental amalgam, and inadequate infrastructure and skilled personnel at the primary care level. These issues were increasingly recognized through the Fifth National Oral Health Survey and routine HMIS data.

In the policy stream, viable solutions had been articulated through WHO guidance, regional strategies, and civil society calls for alignment with the Minamata Convention. Innovations such as the use of Cention N, digital radiography, and integration of oral health into maternal care provided tangible and evidence-based alternatives.

The politics stream featured strong leadership from within the Ministry of Health, alignment with the ruling party's broader health reforms, and support from professional bodies. Strategic engagement with development partners and global policy platforms accelerated the momentum. The alignment of these three streams created a “window of opportunity” in 2022 for the comprehensive policy reform to be launched and rapidly implemented.

### 3.8. Implementation Challenges and Lessons Learned

While the reform agenda has been largely successful, several challenges emerged during implementation. A key constraint has been the limited number of trained dental therapists available in rural settings, leading to underutilization of advanced equipment in those regions. Procurement and supply chain delays affected the timely installation of dental units in some districts.

The rollout of the Oral Health in Pregnancy Program faced logistical challenges, including limited funding, uneven regional uptake, and integration issues with RCH services. These experiences underscore the importance of phased scaling, cross-sector coordination, and continuous evaluation to adapt strategies in real-time.

## 4. Discussion

The comprehensive reforms implemented in Tanzania's oral health sector from 2022 to 2024 have marked significant progress in modernizing infrastructure, enhancing service delivery, and improving patient outcomes. The strategic phasing out of dental amalgam, driven by global health and environmental standards, alongside the enhancement of capabilities for dental therapists, has broadened access to quality dental services, particularly for underserved communities. The integration of oral health into primary care settings further underscores a commitment to holistic health approaches, while the establishment of the Oral Health Scorecard offers a robust mechanism for continuous evaluation and improvement. These measures ensure that the sector's advances are not only significant but also sustainable, demonstrating a model for effective health care reform. A key limitation of this evaluation is that while service-level indicators such as equipment deployment, utilization trends, and treatment ratios demonstrate promising progress, population-level oral health outcomes have not yet been assessed. Longitudinal data on caries and periodontal disease prevalence remain necessary to establish the true impact of these reforms at the population level. Another important dimension for future research is patient perspectives. Incorporating user satisfaction surveys or qualitative interviews would provide valuable insights into community acceptance, perceived benefits, and trust in the reformed oral health system. Although this study relied on DHIS2 data, we were unable to disaggregate access or service utilization by key equity markers such as rural versus urban or socioeconomic status. Future analyses should incorporate disaggregated data to better assess whether reforms have equitably improved access across diverse regions and populations.

Looking ahead, the focus must remain on expanding the impact and reach of these reforms. The potential exploration of tele-dentistry could bring specialized dental care to remote areas, ensuring equitable health service distribution across urban and rural settings [16, 17]. Additionally, fostering public–private partnerships could provide crucial support in terms of funding and expertise, further enhancing the sector's capacity. Alongside these technological and collaborative expansions, there is a pressing need for continued public education campaigns to reduce the prevalence of oral diseases [18, 19]. Such campaigns should emphasize prevention and early treatment, targeting risk factors like sugar consumption and tobacco use [20–22]. By maintaining a proactive and integrated approach to oral health, Tanzania can continue to build on the current reforms, ensuring long-term benefits and setting a precedent for national health care strategies.

Tanzania's comprehensive oral health reforms are notable not only for their scope but also for their alignment with global health policies. Similar initiatives, such as Rwanda's National School oral health screening and India's Amalgam-Free Dentistry program, provide comparative benchmarks, yet few countries have undertaken a multicomponent nationwide reform within a 2-year period. This positions Tanzania as a model for LMICs seeking to operationalize the WHO Global Oral Health Action Plan 2022–2030 and the WHO AFRO regional strategies. The integration of oral health into PHC and maternal services reflects a best-practice model of holistic care and intersectoral policy alignment. Since oral health services have been integrated into antenatal care, future collaboration with maternal and child health programs could enable monitoring of outcomes such as low birth weight, preterm delivery, and maternal infections. Linking oral health data with maternal–child indicators would strengthen the evidence base on the systemic benefits of integration.

## 5. Conclusion

The oral health sector reforms initiated by the Tanzanian government from 2022 to 2024 represent a transformative journey towards improving dental care across the nation. These changes have not only aligned with international health standards by phasing out dental amalgam but have also substantially upgraded the infrastructure and broadened access to dental services. The successful implementation of the Oral Health Scorecard and the expansion of training for dental therapists are testament to Tanzania's commitment to enhancing oral health care. Moving forward, the focus on innovations such as tele-dentistry and strengthening public–private partnerships will be crucial in sustaining these improvements and ensuring that all Tanzanians benefit from high-quality oral health services. This forward-thinking approach sets a commendable example for other nations striving to enhance their healthcare systems and demonstrates the power of integrated and strategic health planning. Future work should include a post-reform national oral health survey to evaluate changes in caries and periodontal disease prevalence. Such evidence will be critical in validating whether service-level improvements translate into measurable gains in population oral health outcomes.

## Data Availability

The data that support the findings of this study are available upon request from the corresponding author. The data are not publicly available due to privacy or ethical restrictions.

## References

[1] World Health Organization (2019). Oral Health | WHO | Regional Office for Africa [Internet]. https://www.afro.who.int/health-topics/oral-health.

[2] World Health Organization (2023). Oral Health. Global Strategy on Oral Health.

[3] Opoku P., Salu S., Azornu C. K., Komesuor J. (2024). Oral Health Knowledge, Practice and Associated Factors Among Junior High School Students of Koforidua, Ghana: A Cross-Sectional Study. *BMC Oral Health*.

[4] Wolf T. G., Cagetti M. G., Fisher J.-M., Seeberger G. K., Campus G. (2021). Non-Communicable Diseases and Oral Health: An Overview. *Frontiers in Oral Health*.

[5] Edward M., Agyapong D., Paul I. K. (2024). Oral Health in Tanzania: Unmasking Its Neglected Dimension. *Public Health Challenges*.

[6] Bensel T., Megiroo S., Kronenberg W., Bömicke W., Ulrichs T., Hinz S. (2024). Oral Health Status of Healthcare Workers in Ilembula/Tanzania During the COVID-19 Condition. *Healthcare*.

[7] Masalu J. R., Mtaya M., Mbawalla H., Nyamuryekung’e K. (2020). The Fifth Tanzania National Oral Health Survey Report. https://hssrc.tamisemi.go.tz/storage/app/uploads/public/61e/962/c04/61e962c047b75470406472.pdf.

[8] Mbawalla H. S., Nyamuryekung’e K. K., Mtaya-Mlangwa M., Masalu J.-R. (2023). Dental Caries Pattern Amongst Tanzanian Children: National Oral Health Survey. *International Dental Journal*.

[9] Tibau A. V., Grube B. D. (2023). Dental Amalgam and the Minamata Convention on Mercury Treaty: Make Mercury History for All. *Journal of Oral & Dental Health*.

[10] United Nations Environment Programme (2013). Minamata Convention on Mercury. https://www.unep.org.

[11] Ministry of Health (2022). Phasing Out the Use of Dental Amalgam in Dental Practice. The United Republic of Tanzania. https://environmentalmedicine.eu/wp-content/uploads/PHASING-OUT-ON-DENTAL-AMALGAM-USE-Tanzania.pdf.

[12] Ministry of Health, Tanzania (2022). Health Management Information System: Implementation Guidelines. https://www.tanzaniahealth.go.tz.

[13] District Health Information System-2 (DHIS2) (2022). *Oral Health Scorecard Development Framework*.

[14] MCW Global (2024). MC-Tanzania’s Dental Therapist Outreach Training Program: Enhancing Community-Based Dentistry in Tanzania. https://mcwglobal.org/mc-tanzanias-dental-therapist-outreach-training-program-enhancing-community-based-dentistry-in-tanzania/.

[15] Ministry of Health, Tanzania (2016). Dental Therapist Training Guide and Practical Guide: Training Pack. https://bridge2aid.org/wp-content/uploads/2016/08/DTP-Section-5-Ministry-of-Health-Training-Guide-and-Practical-GuideTraining-Pack-Read-only.pdf.

[16] Aldosari M., Mendes S. D. R., Aldosari A., Aldosari A., de Abreu M. H. N. G. (2021). Factors Associated With Oral Pain and Oral Health-Related Productivity Loss in the USA, National Health and Nutrition Examination Surveys (NHANES), 2015–2018. *PLoS ONE*.

[17] Mutashar M. T., Abdullah B. H. (2023). Teledentistry for Underserved Populations: An Evidence-Based Exploration of Access, Outcomes, and Implications. *Journal of Research in Medical and Dental Science*.

[18] Malpe M., Choudhari S. G., Nagtode N., Muntode Gharde P. (2024). Beyond the Chair: Exploring the Boundaries of Teledentistry. *Cureus*.

[19] Petersen P. E., Baez R. J., Ogawa H. (2020). Global Application of Oral Disease Prevention and Health Promotion as measured 10 Years After the 2007 World Health Assembly Statement on Oral Health. *Community Dentistry and Oral Epidemiology*.

[20] de Lara J. V. I., Frazão P. (2021). Oral Health Guidelines in the Primary Care Policies of Five Selected Countries: An Integrative Review. *Health Policy OPEN*.

[21] Pallangyo P., Komba M. S., Mkojera Z. S. (2024). Perspectives for the Prevention of Noncommunicable Diseases in Tanzania: Is Knowledge Translated Into Practice?. *Risk Management and Healthcare Policy*.

[22] Fisher J., Varenne B., Narvaez D., Vickers C. (2018). The Minamata Convention and the Phase Down of Dental Amalgam. *Bulletin of the World Health Organization*.

